# Wnt signaling in age-related macular degeneration: human macular tissue and mouse model

**DOI:** 10.1186/s12967-015-0683-x

**Published:** 2015-10-17

**Authors:** Jingsheng Tuo, Yujuan Wang, Rui Cheng, Yichao Li, Mei Chen, Fangfang Qiu, Haohua Qian, Defen Shen, Rosana Penalva, Heping Xu, Jian-Xing Ma, Chi-Chao Chan

**Affiliations:** Laboratory of Immunology, National Eye Institute, National Institutes of Health, 10 Center Drive, Bldg. 10, Rm. 10N103, NIH/NEI, Bethesda, MD 20892-1857 USA; Department of Physiology, University of Oklahoma Health Sciences Center, Oklahoma City, OK USA; Visual Function Core, National Eye Institute, National Institutes of Health, Bethesda, MD USA; Centre for Experimental Medicine, Queen’s University Belfast, Belfast, UK

**Keywords:** Wnt signaling, Antibody against Wnt receptor LRP6, Mouse model, Retinal lesion, *Ccl2*^−*/*−^*/Cx3cr1*^−*/*−^*/rd8* mouse, *Ccl2*^−*/*−^*/Cx3cr1*^*gfp/gfp*^ mouse, Serum kallistatin

## Abstract

**Background:**

The wingless-type MMTV integration site (Wnt) signaling is a group of signal transduction pathways. In canonical Wnt pathway, Wnt ligands bind to low-density lipoprotein receptor-related protein 5 or 6 (LRP5 or LRP6), resulting in phosphorylation and activation of the receptor. We hypothesize that canonical Wnt pathway plays a role in the retinal lesion of age-related macular degeneration (AMD), a leading cause of irreversible central visual loss in elderly.

**Methods:**

We examined LRP6 phosphorylation and Wnt signaling cascade in human retinal sections and plasma kallistatin, an endogenous inhibitor of the Wnt pathway in AMD patients and non-AMD subjects. We also used the *Ccl2*^−*/*−^*/Cx3cr1*^−*/*−^*/rd8* and *Ccl2*^−*/*−^*/Cx3cr1*^*gfp/gfp*^ mouse models with AMD-like retinal degeneration to further explore the involvement of Wnt signaling activation in the retinal lesions in those models and to preclinically evaluate the role of Wnt signaling suppression as a potential therapeutic option for AMD.

**Results:**

We found higher levels of LRP6 (a key Wnt signaling receptor) protein phosphorylation and transcripts of the Wnt pathway-targeted genes, as well as higher beta-catenin protein in AMD macula compared to controls. Kallistatin was decreased in the plasma of AMD patients. Retinal non-phosphorylated-β-catenin and phosphorylated-LRP6 were higher in *Ccl2*^−*/*−^*/Cx3cr1*^−*/*−^*/rd8* mice than that in wild type. Intravitreal administration of an anti-LRP6 antibody slowed the progression of retinal lesions in *Ccl2*^−*/*−^*/Cx3cr1*^−*/*−^*/rd8* and *Ccl2*^−*/*−^*/Cx3cr1*^*gfp/gfp*^ mice. Electroretinography of treated eyes exhibited larger amplitudes compared to controls in both mouse models. A2E, a retinoid byproduct associated with AMD was lower in the treated eyes of *Ccl2*^−*/*−^*/Cx3cr1*^−*/*−^*/rd8* mice. Anti-LRP6 also suppressed the expression of *Tnf*-*α* and *Icam*-*1* in *Ccl2*^−*/*−^*/Cx3cr1*^−*/*−^*/rd8* retinas.

**Conclusions:**

Wnt signaling may be disturbed in AMD patients, which could contribute to the retinal inflammation and increased A2E levels found in AMD. Aberrant activation of canonical Wnt signaling might also contribute to the focal retinal degenerative lesions of mouse models with *Ccl2* and *Cx3cr1* deficiency, and intravitreal administration of anti-LRP6 antibody could be beneficial by deactivating the canonical Wnt pathway.

**Electronic supplementary material:**

The online version of this article (doi:10.1186/s12967-015-0683-x) contains supplementary material, which is available to authorized users.

## Background

Age-related macular degeneration (AMD) is a common cause of irreversible central blindness in the elderly [1]. Pathological features of AMD include degeneration and/or atrophy of both photoreceptors and retinal pigment epithelia (RPE) in the macula. More advanced stages of AMD present as the exudative/neovascular or “wet” form featuring choroidal neovascularization (CNV) and the geographic atrophy or “dry” form featuring significant loss of the photoreceptors and RPE [2]. Even though it is known that various pathways such as inflammation, apoptosis, and pathological angiogenesis are involved during the end stage of the disease [2, 3], the molecular mechanisms that lead to the death of photoreceptors and other retinal cells in AMD remain poorly understood.

The wingless-type MMTV integration site (Wnt) signaling is a group of signal transduction pathways including the canonical pathway, the noncanonical planar cell polarity pathway, and the noncanonical Wnt/calcium pathway [4]. In the canonical Wnt pathway, Wnt ligands bind to frizzled (Fz) receptors or to the coreceptor complex of Fz and low-density lipoprotein receptor-related protein 5 or 6 (LRP5 or LRP6), resulting in phosphorylation and activation of the receptor [5, 6]. Upon activation of the receptor, a signaling cascade is triggered, leading to attenuation of phosphorylation of transcription factor β-catenin and its nuclear translocation [4]. Consequently, β-catenin recruits TCF/LEF transcription factors in the nucleus and stimulates the expression of Wnt target genes including CYCLIN D, c-MYC, AXIN 2, VEGF, ICAM-1, CTGF, TNF-α, and HIF-1. Improper activation of Wnt signaling has been implicated in many pathophysiological conditions including cancer, neurological diseases, and diabetes [4]. Previous reports show that the Wnt signaling pathway is activated in the retinas of laser-induced CNV mouse model, a classic exudative AMD model. The therapeutic potential of blocking Wnt signaling by anti-LRP6 antibody in this model was explored [7]. However, the role of Wnt signaling in dry AMD has not been documented.

We have reported that genetically engineered *Ccl2*^−*/*−^*/Cx3cr1*^−*/*−^ mice on *rd8* background (*Ccl2*^−*/*−^*/Cx3cr1*^−*/*−^*/rd8*) develop a broad spectrum of dry AMD-like pathology with early onset and high penetrance [8–10]. This strain exhibits 100 % penetrance and more prominent RPE and photoreceptor focal degeneration at an earlier age compared to the *rd8* mouse with a single base deletion in the *Crb1* gene [9, 11]. Although retinal dystrophy/dysplasia *rd8* lesions were found mainly in the outer plexiform layer, this double knockout strain also develops pathological features similar to human AMD. These features included deep focal retinal degeneration, which progress with age, photoreceptor thinning and loss, RPE alteration, degeneration and atrophy, and A2E accumulation. A few mice also develop CNV. Using this model, we have successfully demonstrated beneficial effects of long-term dietary intake of long chain omega-3 polyunsaturated fatty acids (n-3) and the Age-Related Eye Disease Study 2 (AREDS2) diet. We also identified the therapeutic efficiency by using an adeno-associated virus vector overexpressing the soluble VEGF receptor gene to trap excess VEGFA and recombinant TSG6 protein (an anti-inflammatory protein produced by mesenchymal stem cells) to alleviate the retinal lesions [12–15]. Recently, the featured focal dry AMD-like degenerative retinal lesions *Ccl2*^−*/*−^*/Cx3cr1*^*gfp/gfp*^ mice without *rd8* background were reported to develop with late-onset, especially after long term blue light exposure [16]. This mouse strain also developed severe RPE degeneration (Additional file 1: Figure S1).

In this study, we examined LRP6 phosphorylation and Wnt signaling cascade in human retinal sections and plasma kallistatin, an endogenous inhibitor of the Wnt pathway in AMD and non-AMD subjects. We also used the *Ccl2*^−*/*−^*/Cx3cr1*^−*/*−^*/rd8* and the *Ccl2*^−*/*−^*/Cx3cr1*^*gfp/gfp*^ without *rd8* mutation murine models to further explore the involvement of Wnt signaling activation in the retinal lesions in those models and to preclinically evaluate the role of Wnt signaling suppression as a potential therapeutic option for AMD.

## Methods

### Ethics statement

This research adhered to the tenets of the Declaration of Helsinki. Each participant provided signed informed consent in accordance with protocols for human subject recruitment and clinical evaluation approved by the Institutional Review and Ethics Boards of the National Eye Institute. The animal studies were conducted in compliance with the ARVO statement for the use of animals, and all animal experiments were performed under protocols approved by the Institutional Animal Care and Use Committees of National Eye Institute, National Institutes of Health, USA; and Queen’s University Belfast, Belfast, UK.

### Immunohistochemistry and mRNA expression of human ocular sections

Archived paraffin ocular sections from 5 AMD cases (3 wet, 2 dry) and 5 age-matched normal eyes were subjected to avidin–biotin-complex immunoperoxidase staining. Endogenous peroxidase activity was blocked in hydrogen peroxide. Polyclonal antihuman goat IgG antibodies against total LRP6 (Cell Signaling Technology, Danvers, MA) were applied at a 1:50 dilution for 60 min at room temperature. Biotinylated rabbit anti-goat IgG was used as the linker molecule between the LRP6 antibody and avidin–biotin-peroxidase complex for 60 min before application of the ABC complex (Vector Lab, Mountain View, CA, USA). Diaminobenzidine-hydrogen peroxide was used as the chromogen. The sections were counterstained with 1 % methyl green. The staining was evaluated based the intensity of brown-blackish color on the sections. For β-catenin immunohistochemistry, the antibody was antihuman rabbit IgG antibody (sc-7199, Santa Cruz Biotechnology, Inc. Dallas, TX, USA).

The AMD cases and age-matched normal eyes were used for transcript analysis. Neuroretinal and RPE cells, Bruch’s membrane and choroidal capillaries in the macular area from each ocular section were microdissected for RNA extraction using the Paradise™ Sample Quality Assessment Kit (Arcturus Bioscience), followed by cDNA synthesis using Invitrogen SuperScript™ II reverse transcriptase (Invitrogen, Carlsbad, CA). A260/A280 of the RNA was greater or equal to 1.80, indicating that the RNA was pure. Human Total RNA (Life Technology, Gaithersburg, MD, USA) was converted to cDNA and used as the control for RT-PCR reactions. The primers for SYBR Green qPCR were synthesized by SABiosciences and supplied in the Real-Time RT-PCR Gene Expression Analysis kit (SABiosciences, Frederick, MD, USA). Following PCR, a thermal melt profile was performed for amplicon identification. Folder changes (2^−∆∆Ct^ analysis method) were calculated by comparing with Ct values obtained from a paralleled amplification of Human Total RNA.

### Human plasma for ELISA quantification of Kallistatin

The study group demographics are summarized in Table 1. Participant selection and clinical evaluation have been previously defined [17–20]. AMD cases were all in advanced stages and the controls presented either no drusen or fewer than 5 small drusen (<63 µm in diameter) and no signs of other retinal diseases, including but not limited to high myopia, retinal dystrophies, central serous retinopathy, vein occlusion, diabetic retinopathy, or uveitis. The patients and controls were all self-identified as Caucasian and non-Hispanic.

**Table 1:** Human plasma samples

	Number	Average age	Male (%)	Female	Wet	Dry
AMD	66	75.7 ± 10.4	28 (42.4)	38	56	10
Control	53	63.4 ± 8.9	15 (28.3)	38	–	–

Kallistatin levels in human plasma were quantified by ELISA (R&D Systems, Minneapolis, MN, USA), as previously described [21]. Briefly, 96 well microplates were coated with mouse anti-human kallistatin capture antibody overnight at room temperature (RT). Nonspecific binding to capture antibody was blocking by 1 % BSA in PBS at RT for 1 h. Plasma samples were diluted (1/20,000) with 1 % BSA in PBS. Samples and standards were added to wells coated with mouse anti-human kallistatin capture antibody. After incubation at RT for 2 h and the extensive washing with PBS with Tween-20, the plate was incubated with 100 μL biotinylated goat anti-human kallistatin detection antibody for 2 h, followed by incubation with 100 μL Streptavidin conjugated to horseradish-peroxidase (R&D Systems) for 20 min. After the addition of H_2_O_2_/tetramethylbenzidine for 20 min, 50 μL 2 N H_2_SO_4_ was added to stop the reaction. Plate was read immediately at 450 nm (VICTOR3 V™ Multilabel Counter, PerkinElmer Life And Analytical Sciences, Inc, Waltham, MA, USA). The personnel testing the samples were blind to the information and samples were identified by numeric codes. All measurements were performed in triplicate for each sample, and the mean values were calculated. Inter- and intra-assay variations were <5 and 10 %, respectively.

### Wnt pathway component mRNA array

Total RNA from mouse retina/RPE was extracted using Trizol (Invitrogen, Carlsbad, CA, USA). cDNA was synthesized using reverse transcriptase (Invitrogen). The array to identify possible differences in the ocular expression of Wnt-related genes between *Ccl2*^−*/*−^*/Cx3cr1*^−*/*−^*/rd8* and wild type B6 N mice was performed using Mouse WNT Signaling Pathway RT^2^ Profiler™ PCR Arrays according to the manufacturer’s protocols (Qiagen/SABiosciences, Gaithersburg, MD, USA). The plate contains assays for 92 Wnt pathway associated genes and 4 assays to candidate endogenous control genes (Additional file 2: Table S1). The reaction was run on an ABI 7500 System (Applied Biosystems, CA, USA). Expression values were determined with DataAssist™ v2.0 Software (Applied Biosystems).

### Animals and treatment

The animal study was conducted in compliance with the ARVO statement for the use of animals, and all animal experiments were performed under protocols approved by the Institutional Animal Care and Use Committees of National Eye Institute, National Institutes of Health, USA and Queen’s University, Belfast, UK. Mice were originated and bred from National Eye Institute, National Institutes of Health, USA (*Ccl2*^−*/*−^*/Cx3cr1*^−*/*−^*/rd8*) or Queen’s University, Belfast, UK (*Ccl2*^−*/*−^*/Cx3cr1*^*gfp/gfp*^).

The generation of *Ccl2*^−*/*−^*/Cx3cr1*^−*/*−^ mice on *rd8* background (*Ccl2*^−*/*−^*/Cx3cr1*^−*/*−^*/rd8*) and *Ccl2*^−*/*−^*/Cx3cr1*^−*/*−^ without *rd8* background (*Ccl2*^−*/*−^*/Cx3cr1*^*gfp/gfp*^) and their pathological features have been reported previously [10, 16]. Specifically, both mouse strains were generated as a model of progressive, focal retinal degeneration that mimics certain features of human AMD lesions, predominant dry AMD phenotype [10, 16]. *Ccl2*^−*/*−^*/Cx3cr1*^−*/*−^*/rd8* develops retinal lesions spontaneously while the retinal lesions in *Ccl2*^−*/*−^*/Cx3cr1*^*gfp/gfp*^ can be induced by exposing of 3-month old mice to blue light (465 nm) for 8 h/day for 6 months. The monoclonal anti-LRP6 antibody was obtained using a recombinant peptide comprising the E1E2 domain of murine LRP6 as the antigen [7]. Nonspecific mouse IgG (mIgG) purchased from Vector Laboratories (Burlingame, CA, USA) was used as a control for anti-LRP6.

Single injections of 500 ng of anti-LRP6 in 1 μL volume were intravitreally administered to the right eyes of 6-week-old *Ccl2*^−*/*−^*/Cx3cr1*^−*/*−^*/rd8*; the contralateral eye was injected with 500 ng of mIgG in the same volume as a control (n = 35). The same amount of anti-LRP6 was intravitreally injected once per month into one eye of 6-month-old *Ccl2*^−*/*−^*/Cx3cr1*^*gfp/gfp*^ (i.e. after 3 months light exposure, n = 20, 10 for treatment and 10 for control). The control eyes were intravitreally given 500 ng (1 μL) mIgG once every month. Eyes were harvested at 2 months (*Ccl2*^−*/*−^*/Cx3cr1*^−*/*−^*/rd8*) or 3 months (*Ccl2*^−*/*−^*/Cx3cr1*^*gfp/gfp*^) after the injection for various measurements. The eyes of C57BL/6N with *rd8* mutations were used for an assay control.

### Fundus photography and scoring

We evaluated the lesion changes by comparing sequential fundus photographs taken in the same fundus area of each eye before the injection and every month after the injection using a Karl Storz veterinary otoendoscope [22]. Progression was defined as a >10 % increase in the number of retinal lesions (Score +1), a >50 % increase in the lesion size in at least one out of three of the lesions (Score +2), more than five fused lesions or the appearance of more than two chorioretinal scars (Score +3), and diffuse chorioretinal scars (Score +4) compared to baseline. Regression was defined as a >10 % decrease in the number of retinal lesions (Score −1), a >50 % decrease in lesion size in at least one out of three of the lesions (Score −2), a >50 % disappearance of retinal lesions (Score −3) or a total disappearance of retinal lesions (Score −4). To avoid bias, a masked observer evaluated all fundus photographs. The lesion scores of each eye were estimated by cross-comparison of the same area based on above criteria. Fundus photography taken from the mice tested in Belfast site used the topic endoscopic fundus imaging (TEFI) system described previously [22, 23].

### Electroretinography (ERG)

Light-adapted ERG responses for *Ccl2*^−*/*−^*/Cx3cr1*^−*/*−^*/rd8* were used to evaluate cone-pathway function in the retina following a published procedure [24]. Briefly, mice adapted to room light were anesthetized with i.p. injection of a mixture containing ketamine and xylazine. Pupils were dilated with 2.5 % phenylephrine (Alcon Inc, Fort Worth, TX, USA) and 1 % tropicamide (Alcon Inc), and cornea was anesthetized with 0.5 % proparacaine (Alcon Inc). Animals were placed on a heating pad to maintain body temperature. ERG responses were recorded with gold-wire electrodes placed on the center of the cornea and measured using a commercial Espion E2 system (Diagnosys LLC, Lowell, MA, USA). Light emitting diodes with built in ultraviolet (UV) colordome provide ganzfeld UV (365 nm) or green (514 nm) light stimulus. Photopic ERG responses were recorded in the presence of a rod-saturating background of 20 sc cd/m^2^. The dark-adapted ERG was recorded for *Ccl2*^−*/*−^*/Cx3cr1*^*gfp/gfp*^ (LKC Technologies, Gaithersburg, MD, USA) [25].

### Histology

Whole mouse eyes were fixed in 4 % glutaraldehyde-10 % formalin and then embedded in methacrylate. The eyes were serially sectioned in the vertical pupillary-optic nerve plane. Each eye was cut into 6 sections. All sections were stained with hematoxylin and eosin. If an ocular lesion was observed, another 6-12 sections were cut through the lesion. These slides were also stained with Periodic Acid Schiff (PAS) to highlight the Bruch’s membrane and the basement membrane of small neovascular vessels.

### Retinal lipofuscin extraction and quantification

A2E ([2,6-dimethyl-8-(2,6,6-trimethyl-1-cyclohexen-1-yl)-1E,3E,5E,7E-octatetra-enyl]-1-(2-hydroxyethyl)-4-[4-methyl-6(2,6,6-trimethyl-1-cyclohexen-1-yl) 1E,3E,5E,7E-hexatrienyl]-pyridinium) is the major component of lipofuscin fluorophores generated from the visual cycle flux of all-trans-retinol. The molecule is particularly correlated with retinal aging and AMD pathogenesis [26]. A2E was extracted via chloroform/methanol as previously described [27]. The extracts dissolved in methanol were separated by HPLC (Agilent 1100 LC, Wilmington, DE, USA) and detected by an ultraviolet detector at a wavelength of 435 nm. A2E was quantified using external A2E standards [28].

### Western blot analysis

Western blot analysis detected the phosphorylated level of LRP6. Mouse retina was dissected and homogenized for protein extraction. Equal amounts (50 μg) of total protein from each sample were resolved by SDS polyacrylamide gel electrophoresis (SDS-PAGE) and transferred onto a nitrocellulose membrane. The membrane was blocked with 5 % nonfat milk and separately blotted with primary antibodies (anti-Non-phosphorylated β-Catenin, Cell Signaling Technology; anti-phosphorylated LRP6, Cell Signaling Technology; and anti-LRP6, self-made). After thorough washes, the membrane was incubated with the peroxidase-conjugated horse anti-rabbit or horse anti-mouse antibody (Vector Lab). The signal was developed with the enhanced chemiluminescence (ECL) system (Pierce, Rockford, IL, USA). Membranes were stripped and re-blotted with anti-β-actin (Sigma-Aldrich, St. Louis, MO, USA) as the loading control. Densitometry of the signal bands on digital images was quantified using FluorChem Q software (ProteinSimple, Santa Clara, CA, USA).

### Quantification of mRNA expression in mouse eyes by RT-PCR

cDNA synthesis was described as above. The primers/probes were purchased from Applied Biosystems as inventoried TaqMan gene expression reagents. Relative quantitative real time PCR was performed according to ABI’s instructions. To determine the Ct values, the threshold level of fluorescence was set manually in the early phase of PCR amplification. ABI SDS 1.3.1 software and the 2^−ΔΔCt^ analysis method were used to determine relative amounts of product using *Gapdh* as an endogenous control. Each sample was analyzed in triplicate.

### Statistical analysis

Multiple means were compared by paired T-test. Differences were considered significant when *p* <0.05. The collective ERG amplitudes were calculated by the area under curve (AUC) and compared by T-test between the groups. Comparison of plasma kallistatin levels between AMD patients were performed by independent-samples T test.

## Results

### Aberrant expression of Wnt signaling in human AMD

**Fig. 1:**
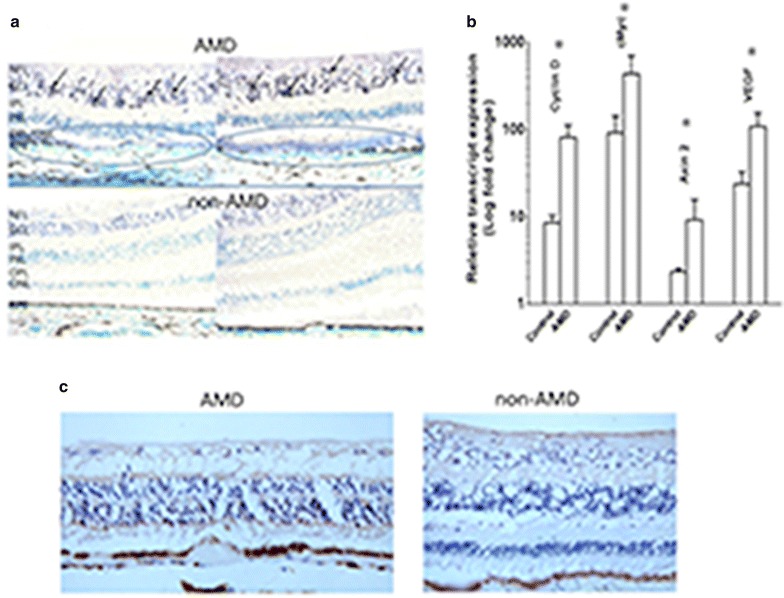
Wnt signaling molecules in human retina. **a** Immunochemistry of phosphorylated LRP6 found higher activated LRP6 in the ganglion cell layer (*brown-blackish staining*, *arrows*) of the retinal sections of AMD maculae (*upper panel*) than in non-AMD macular tissues (*bottom panel*). Atrophic/degenerative photoreceptors and abnormal RPE cells are also noted in the macula of AMD specimens (*blue circles*). These areas also illustrate stronger immunoreactivities compared to the normal macula in the lower panel. *NFL* nerve fiber layer, *GCL* ganglion cell layer, *IPL* inner plexiform layer, *INL* inner nuclear layer, *OPL* outer plexiform layer, *ONL* outer nuclear layer. **b** mRNA expression of microdissected macular cells from human retinal section was measured by RT-PCR. Higher expression of *CYCLIN D*, *cMYC*, *AXIN2*, and *VEGF* was observed in AMD patients compared to non-AMD controls (N = 5, *Error bar* mean ± SEM); *p < 0.05. **c** Immunochmistry of β-catenin in the macula. Higher expression of β-catenin was illustrated in AMD macular region compared to non-AMD macular area

We examined Wnt signaling in AMD using immunohistochemistry of LRP6 expression in human retinal sections. More intense immunoreactivity to LRP6 was found in the retinal and RPE cells of the macular region of the human eyes with AMD compared to normal maculae (Fig. 1a). The specificity of the antibody was confirmed in cell lysate using a human retinal pigment epithelial cell line, ARPE-19 (Additional file 1: Figure S2). Elevated mRNA expression of *CYCLIN D, C*-*MYC, AXIN 2* and *VEGF,* which are the target genes of β-catenin, were also detected in the AMD macular lesions compared to age-matched non-AMD controls (Fig. 1b). Moreover, β-catenin protein expression was also higher in the AMD macular region (Fig. 1c).

**Fig. 2:**
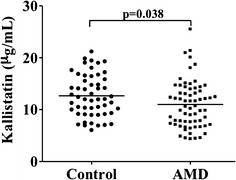
Plasma Kallistatin of human samples. The average plasma kallistatin levels in AMD patients (n = 66) were decreased compared to controls (N = 53, 2 pairs of sample with identical measurements)

To check the Wnt signaling status in larger number of AMD patients, we measured plasma kallistatin, an endogenous inhibitor of the Wnt pathway. Demographic data of the human subjects are summarized in Table 1. Concentrations of kallistatin were significantly decreased in the plasma of AMD patients compared to those from non-AMD subjects. The averages of kallistatin level were 12.67 µg/ml in controls and 10.99 µg/ml in AMD group, demonstrating a 13.3 % less in the AMD in comparison with the controls (t = 2.104, p = 0.038; Fig. 2). There was no correlation between the kallistatin level and age in both the AMD and control groups (Additional file 1: Figure S3), which justifies the exclusion of age as a cofounder in the statistical analysis. Six AMD plasma samples showed higher kallistatin levels, departing from the rest of the AMD cluster (Fig. 2). Clinical information from these 6 cases did not unveil any common features to explain the deviation (Additional file 2: Table S2).

### Manipulation of Wnt/β-catenin is able to rescue the AMD-like lesions of focal retinal degeneration in mouse models

**Fig. 3:**
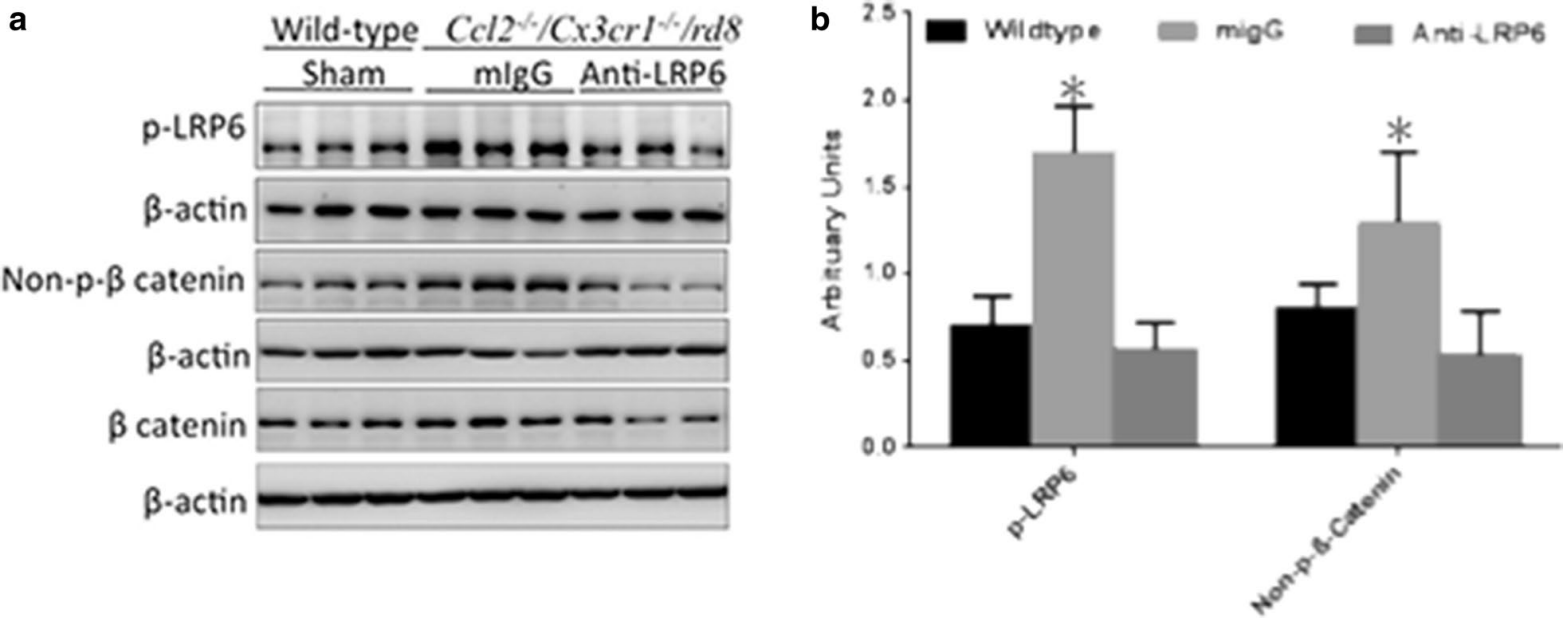
Western blot analysis of key components of the Wnt pathway in mouse retina. **a** Phosphorylated LRP6 and non-phosphorylated β-catenin were higher in the retina of *Ccl2* ^−*/*−^ */Cx3cr1* ^−*/*−^ */rd8* compared to wildtype B6N mice. Phosphorylated LRP6 and non-phosphorylated β-catenin were measured in retinal homogenates of mice treated with the anti-LRP6 antibody or control mIgG. Reduced phosphorylated LRP6 and non-phosphorylated β-catenin were observed after the anti-LRP6 antibody treatment (n = 3 from each condition). **b** Quantitative analysis of 3 experiments, *: <0.05

To test whether the Wnt/β-catenin is activated in C*cl2*^−*/*−^*/Cx3cr1*^−*/*−^*/rd8* mice, the retinal mRNA levels of 92 Wnt pathway genes were measured; however, no significant differences between the expression of these genes in the *Ccl2*^−*/*−^*/Cx3cr1*^−*/*−^*/rd8* mice and wild type B6 N mice (with *rd8* lesions) were found (Additional file 1: Figure S4). At the protein level, phosphorylated LRP6 (p-LRP6) and non-phosphorylated β-catenin (np-b-catenin) were elevated in the retinas of *Ccl2*^−*/*−^*/Cx3cr1*^−*/*−^*/rd8* compared to B6N (Fig. 3a, 3 lanes on the left for B6N and 3 lanes in the middle for *Ccl2*^−*/*−^*/Cx3cr1*^−*/*−^*/rd8*; Fig. 3b, quantitative analysis), suggesting that the Wnt/β-catenin pathway might contribute to the AMD-like lesion.

**Fig. 4:**
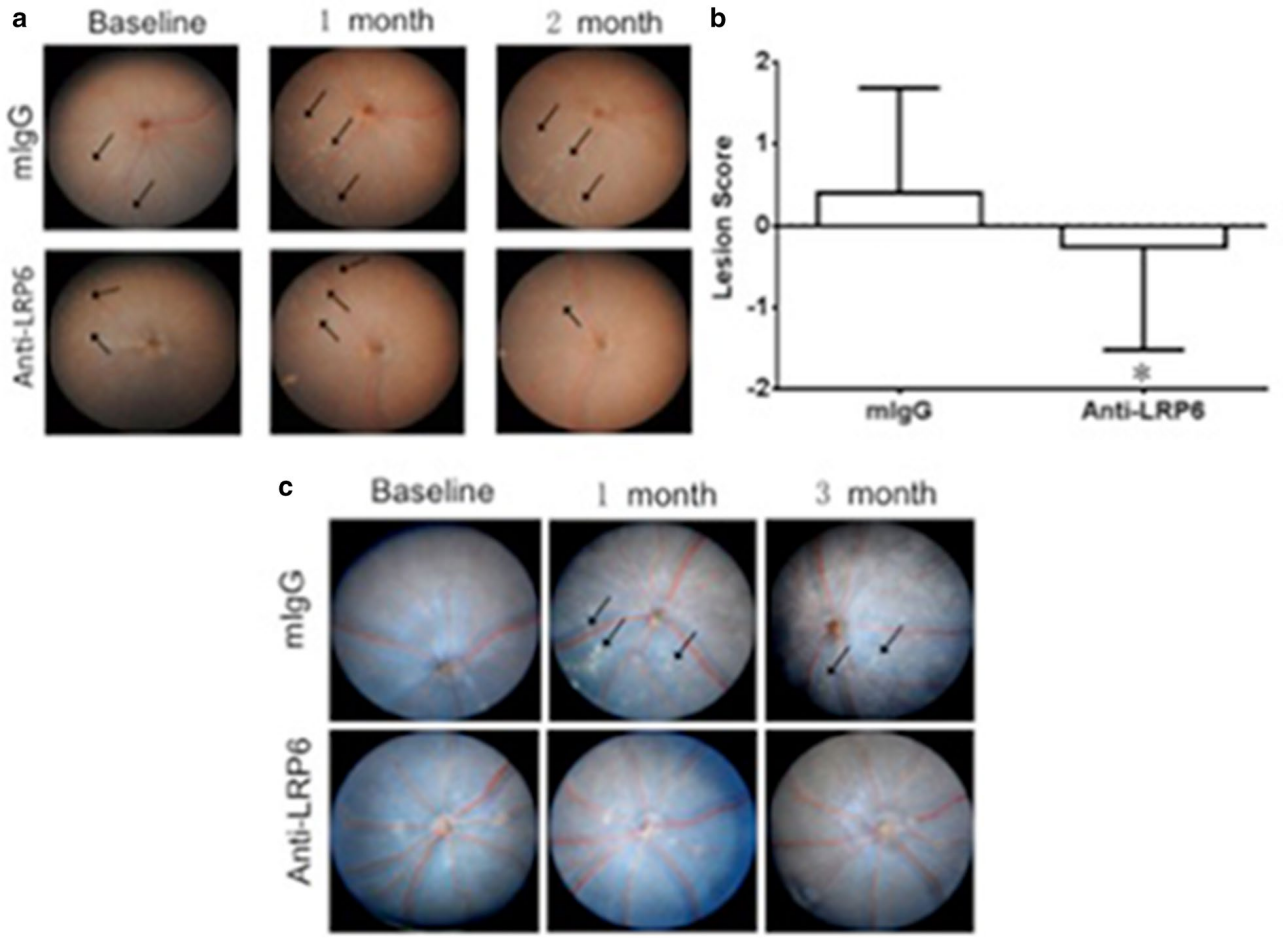
Slower progression or alleviation of retinal lesions in the anti-LRP6 treated eyes as compared to the control in both models. **a** Representative fundoscopic images of *Ccl2* ^−*/*−^ */Cx3cr1* ^−*/*−^ */rd8*. *Arrows* indicate the *yellowish deep* retinal lesions. **b** Comparison of the average retinal lesion scores between treated and untreated eyes of 35 *Ccl2* ^−*/*−^ */Cx3cr1* ^−*/*−^ */rd8* mice. *Error bar* mean ± SEM; *: *p* < 0.01. **c** Representative fundoscopic images of *Ccl2* ^−*/*−^ */Cx3cr1* ^*gfp/gfp*^. *Arrows* indicate the *yellowish deep* retinal lesions. In *Ccl2* ^−*/*−^ */Cx3cr1* ^*gfp/gfp*^, only 5 out of 10 treated eyes but 7 out of 10 control eyes showed lesion progression

We attempted to intervene in the activation of the Wnt signaling pathway by intravitreal administration of anti-LRP6 antibody directly into one eye of our two mouse models of AMD; the contralateral eye was used as sham-injection as a control. Three independent experiments (n = 13 each in the first and second experiments, and n = 9 in the third experiment) were performed in *Ccl2*^−*/*−^*/Cx3cr1*^−*/*−^*/rd8* and yielded reproducible clinical results: funduscopy showed slower progression or alleviation of retinal lesions in the anti-LRP6 antibody-treated eyes compared to the control mIgG-injected eyes in *Ccl2*^−*/*−^*/Cx3cr1*^−*/*−^*/rd8* mice (Fig. 4a), whereas the natural course of lesion in the model is to worsen over time [8, 10]. Among 35 pairs of eyes, 26 (74.3 %) were improved, 8 (22.8 %) stayed the same and 1 (2.9 %) progressed. The retinal lesions scores were significantly different between the treated and untreated eyes (Fig. 4b). The clinical observation of *Ccl2*^−*/*−^*/Cx3cr1*^*gfp/gfp*^ also showed slower progression of retinal lesion after the anti-LRP6 antibody injection (Fig. 4c), a repeatable data compared to that in *Ccl2*^−*/*−^*/Cx3cr1*^−*/*−^*/rd8* mice (Fig. 4a).

**Fig. 5:**
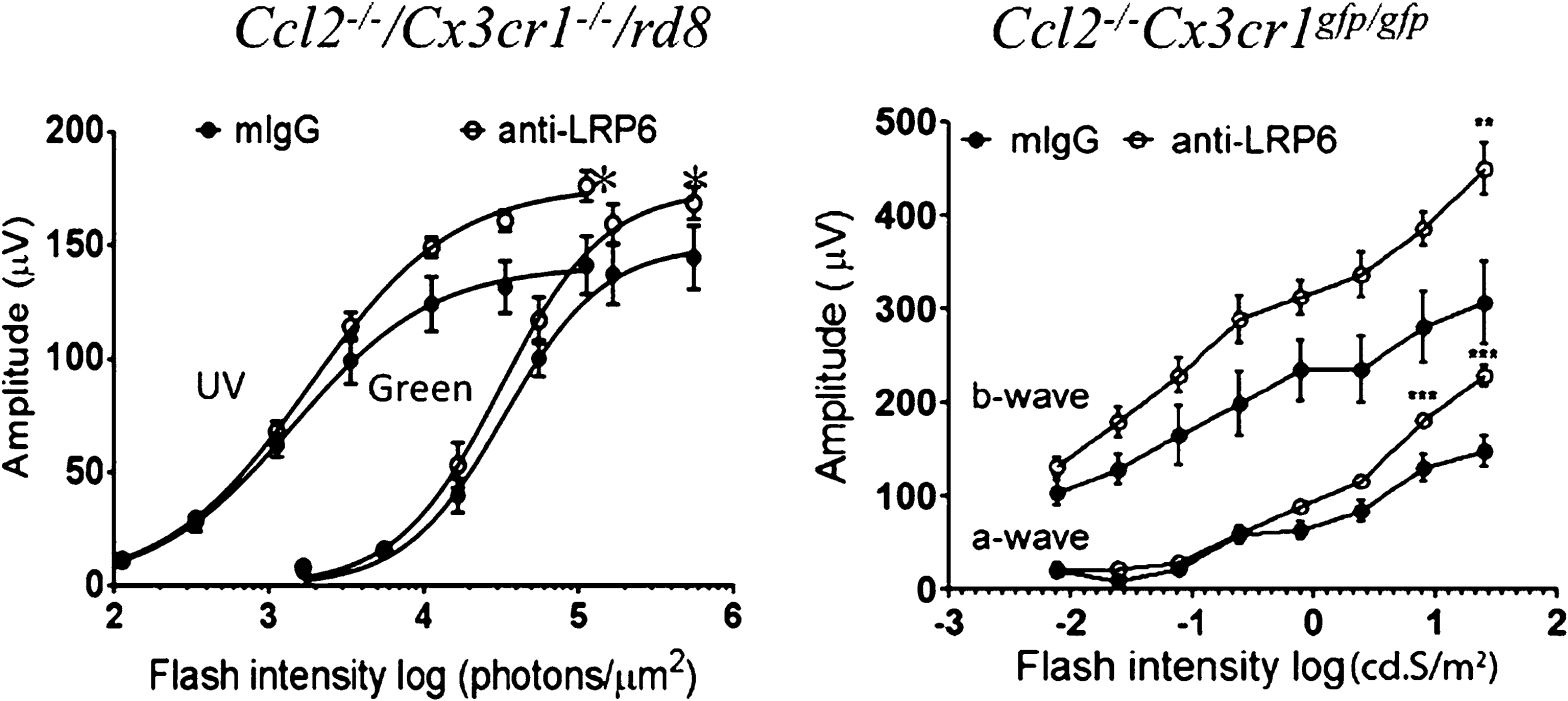
ERG of *Ccl2* ^−*/*−^ */Cx3cr1* ^−*/*−^ */rd8* and *Ccl2* ^−*/*−^ */Cx3cr1* ^*gfp/gfp*^ mice. *Left panel* Averaged photopic b-wave amplitudes elicited with UV and *green light* flashes from the treated and control eyes in *Ccl2* ^−*/*−^ */Cx3cr1* ^−*/*−^ */rd8.* *Right panel* Serial ERG responses to different intensities of white light flashes after dark adaptation were recorded in the treated and control eyes in *Ccl2* ^−*/*−^ */Cx3cr1* ^*gfp/gfp*^. Data are present as mean ± SEM of 5 mice. *: *p* < 0.05; **: *p* < 0.01. ***: *p* < 0.001

We used ERG to test functionality differences in treated and untreated eyes. Because the retinal lesions of *Ccl2*^−*/*−^*/Cx3cr1*^−*/*−^*/rd8* are more abundant in ventral retina (Fig. 4a) where cones preferentially express UV visual pigment, we tested UV flash ERG in light-adapted mice. *Ccl2*^−*/*−^*/Cx3cr1*^−*/*−^*/rd8* mice had lower b wave amplitudes for both UV and green light stimulation than wild type B6 N mice (Additional file 1: Figure S5). Two months after the anti-LRP6 injection, ERG b-wave amplitudes in UV and green light stimulation were enhanced with greater improvements under UV light stimulus in *Ccl2*^−*/*−^*/Cx3cr1*^−*/*−^*/rd8* (Fig. 5 left panel). Even though a different ERG protocol was used for *Ccl2*^−*/*−^*/Cx3cr1*^*gfp/gfp*^, scotopic (dark-adaptation) ERG also exhibited larger amplitudes in both a- and b-waves in the treated eyes compared to control mIgG treated eyes (Fig. 5 right panel).

**Fig. 6:**
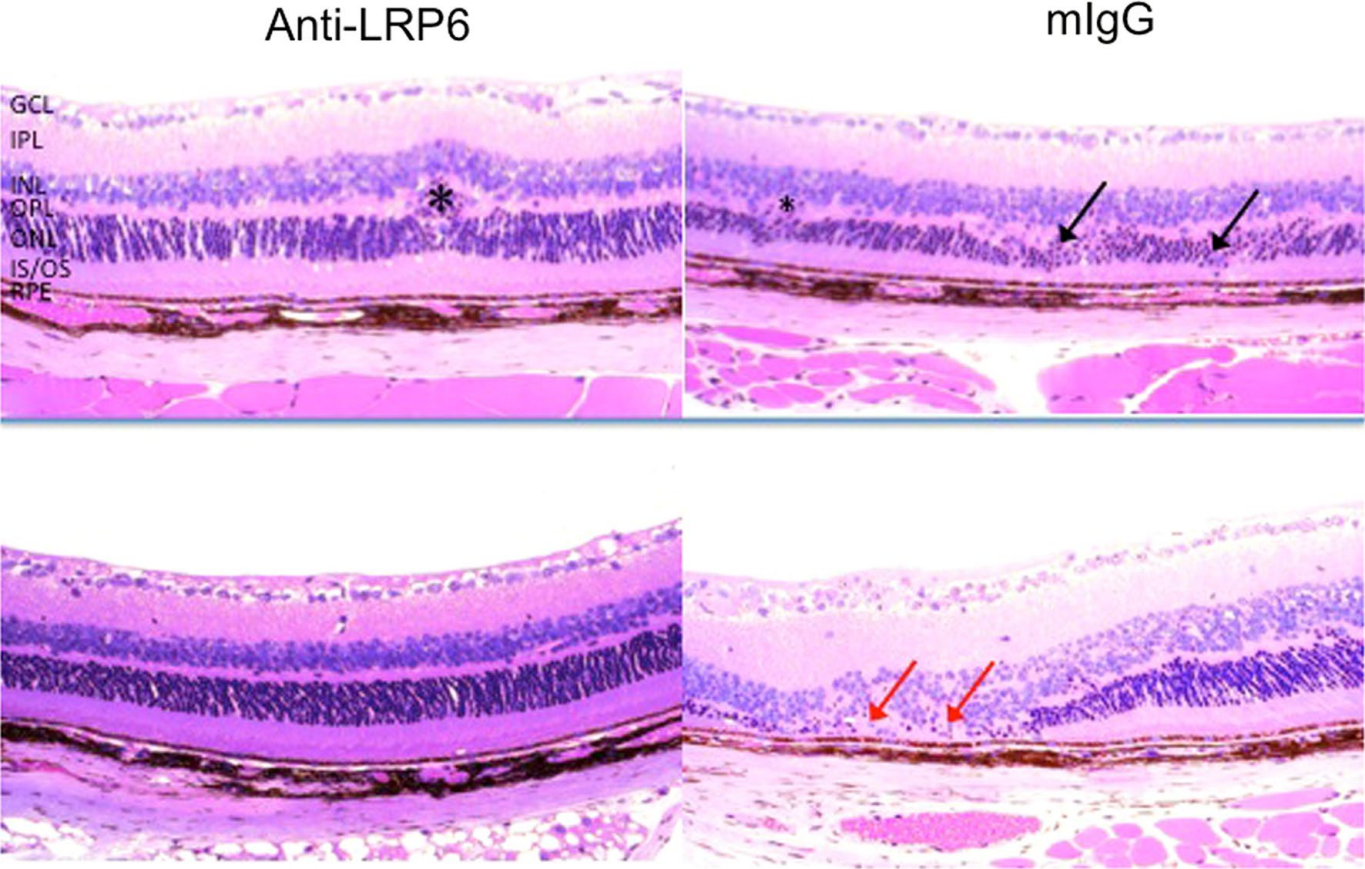
Histology of the *Ccl2* ^−*/*−^ */Cx3cr1* ^−*/*−^ */rd8* mouse retina. Representative histological micrographs: fewer lesions in anti-LRP6 treated eyes, as shown by the reduced loss of photoreceptors and more organized retinal structure. *Black arrows* indicate degenerative photoreceptors in the OPL (*top right panel*). *Red arrows* show degeneration and atrophy of ONL and loss of IS/OS in mIgG-injected group (*bottom right panel*). Some Crb1-associated lesions in the OPL are also seen (*asterisk*, *top left panel*) (H&E, original magnification, ×200). *GCL* ganglion cell layer, *IPL* inner plexiform layer, *INL* inner nuclear layer, *OPL* outer plexiform layer, *ONL* outer nuclear layer, *IS*/*OS* inner segments/outer segments of photoreceptors

**Fig. 7:**
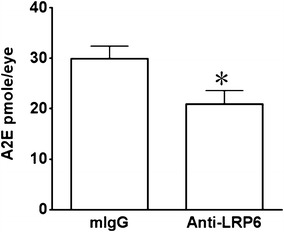
Retinal A2E accumulation in the *Ccl2* ^−*/*−^ */Cx3cr1* ^−*/*−^ */rd8* mice. Retinal A2E levels in anti-LRP6 treated and control eyes of *Ccl2* ^−*/*−^ */Cx3cr1* ^−*/*−^ */rd8.* A2E concentrations were determined by HPLC in the retina. The A2E level was reduced after anti-LRP6 antibody injection. n = 5 from each group; *: *p* < 0.05

Histopathologically, the majority of the treated eyes in *Ccl2*^−*/*−^*/Cx3cr1*^−*/*−^*/rd8* showed stable or improvement of retinal lesions 2 months after the injection, which confirmed the clinical observation in *Ccl2*^−*/*−^*/Cx3cr1*^−*/*−^*/rd8.* Anti-LRP6-treated eyes showed better retinal morphology than that in the mIgG-treated group, characterized by less photoreceptor atrophy, smaller retinal lesions, and thicker retinal inner and outer segment layers (Fig. 6). However, retinal dysplasia caused by rd8 was generally unchanged between treated and untreated eyes. Histological examination between the treated and control eyes from 10 pairs of eyes revealed a decreased lesion severity in 8 pairs and similar lesion severity in 2 pairs. At the biochemical level, A2E was decreased in the anti-LRP6-treated eyes compared to mIgG-injected controls in *Ccl2*^−*/*−^*/Cx3cr1*^−*/*−^*/rd8* mice (Fig. 7).

We analyzed the key components of canonic Wnt-β-catenin signaling in paired retinas that showed a clearly improved fundus in the treated eyes when compared to control eyes. Western blot analysis indicated that the anti-LRP6 antibody suppressed the phosphorylation of LRP6 and the dephosphorylation of β-catenin compared to the control mIgG treatment, indicating that this anti-LRP6 antibody blocked the activation of the Wnt signaling pathway (Fig. 3, 3 lanes on the middle and 3 lanes on the right).

**Fig. 8:**
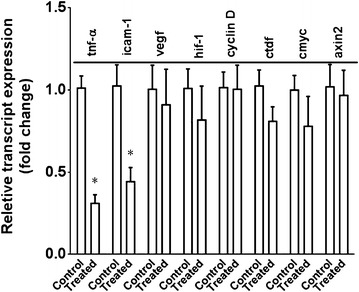
mRNA expression of Wnt signaling pathway target genes in the treated and control eyes in *Ccl2* ^−*/*−^ */Cx3cr1* ^−*/*−^ */rd8* mice. In general, there is lower transcript level in the eyes received anti-LRP6 compared to the control eyes measured by quantitative RT-PCR. *: *p* < 0.05

Quantitative RT-PCR of 5 pairs of *Ccl2*^−*/*−^*/Cx3cr1*^−*/*−^*/rd8* eyes showed decreased *Tnf*-*α* and *Icam*-*1* mRNA; however, *Vegf, Hif*-*1, Cyclin d, Ctdf, c*-*myc,* and *Axin2* transcripts did not change significantly in the retinal tissue after the anti-LRP6 treatment (Fig. 8).

## Discussion

In this study, we found an activated canonical Wnt-β-catenin signaling pathway in human macular tissue from AMD patients and in a murine model with focal retinal degeneration. The notion of the involvement of Wnt signaling in AMD is also supported by the systemic decrease of kallistatin, an endogenous Wnt signaling antagonist, in AMD patients. The blockage of Wnt/β-catenin signaling with locally administered anti-LRP6 antibody arrested the retinal lesion in two murine models with focal retinal AMD-like lesions. The improvement of the progressive degenerative lesions by histology and retained retinal function correlated to suppression of inflammation-related gene expression, particularly *Tnf*-*α* and *Icam*-*1*.

Kallistatin is an endogenous anti-angiogenic and anti-inflammatory factor in the serine proteinase inhibitor (SERPIN) family [29–34]. Previous studies have shown that kallistatin binds to LRP6 and inhibits Wnt signaling, suggesting the Wnt-inhibitory activity is likely responsible for the anti-angiogenic and anti-inflammatory activity of this SERPIN [31, 32]. However, its association with AMD patients has not been established. This study demonstrated for the first time that kallistatin was significantly decreased in the plasma of AMD patients compared to non-AMD subjects. While our sample set differs substantially in the average ages between the AMD and control groups, we did not observe a link between the age and the kallistatin levels in both the AMD and control groups with correlation analysis, which justified the exclusion of age as a confounder in comparing the kallistatin levels between the AMD and controls. Decreased kallistatin levels may contribute to Wnt pathway activation in ocular tissues, leading to inflammation. Further studies are needed to evaluate the possible role of kallistatin in AMD pathogenesis.

The molecular analysis of mouse retinal tissues indicated that the development of lesions correlated with canonical Wnt pathway activation in the retina at the post-translational but not the transcriptional level, as we did not detect altered mRNA expression of Wnt components in an mRNA array but did find altered protein phosphorylation of canonical Wnt/β-catenin proteins. This finding provided the clue for proper targeting of the pathway to manage the retinal lesions. The Wnt pathway is known to mediate various physiological and pathological processes including angiogenesis, fibrosis, and inflammation [35, 36]. Wnt signaling pathway also shows neuroprotective effect during development, such as neuronal migration, synaptic differentiation, mature synapse modulation and synaptic plasticity [37–40]. However, previous study reported that very limited immunoreactivity in the adult retina, suggesting lower basal level of Wnt pathway in adult compared to that in younger retina [41]. Those findings also suggest that the Wnt pathway in adult mice may not be essential for adult neuron survival. Excessive Wnt signaling activation in certain cell types has deleterious effects, including tumorigenesis and angiogenesis [42, 43]. Our study also demonstrates that overexpression of certain Wnt signaling may worsen focal retinal degeneration and AMD lesions.

Multiple Wnt-relevant pathways were activated during advanced AMD, when angiogenesis and inflammation play important roles [44, 45]. However, a parallel study of the above pathways with Wnt cascade in AMD has not been well demonstrated. The retinal lesions in the mouse models used in this study showed disturbances in angiogenesis, oxidative stress, apoptosis, and inflammation [13, 14, 16, 46–49]. We selected inflammatory (TNF-α and ICAM-1), angiogenic (VEGF and HIF), proliferative (CYCLIN D and c-MYC), oxidative stress (c-MYC), fibrotic (CTGF), and Wnt inhibitory (AXIN2) markers to determine the molecular profile in AMD. The results indicate that the suppression of Wnt signaling mainly alters the inflammatory responses.

The Wnt signaling pathway has several intersections with other pathways including inflammatory processes such as TNF-α and NF-kB signaling [36, 50, 51]. *Ccl2*^−*/*−^*/Cx3cr1*^−*/*−^*/rd8* mice express a spectrum of immunological features of AMD, including increased deposition of complement factors, altered secretion of chemokines and cytokines, and the accumulation of activated microglia and macrophages in the retinal lesions [9, 16, 52–54]. The inflammatory profile in this strain seems to result from the deficiency of *Ccl2 and Cx3cr1* rather than the rd8 mutation alone [9, 55]. Although we do not have data directly linking the Wnt activation with the deficiency of *Ccl2* and *Cx3cr1*, the fact that two animal models with these two deletions show similar results supports the involvement and the importance of Wnt signaling pathway in AMD pathogenesis.

This study demonstrates that the Wnt pathway activation is associated with focal retinal degeneration in animal models and human AMD. Blockage of Wnt signaling using a specific antibody for LRP6 improved the retinal structure and function, likely through reducing oxidative stress and inflammation. Significant lower ocular *Tnf*-*α* levels have been measured in the *Ccl2*^−*/*−^*/Cx3cr1*^−*/*−^*/rd8* mouse retina treated with omega-3, AREDS2 diet, TSG-6, PEDF, or Naloxane [12, 14, 15, 46, 47]. This observation further supports the pathogenic role of the Wnt pathway in retinal degeneration in AMD. However, more AMD eyes are needed to verify Wnt signaling activity and conclude about the pathogenic role of Wnt signaling in this disease. Further, kallistatin measurement in AMD patients’ plasma is limited by the cross-sectional study. A longitudinal prospective study of kallistatin levels in different stages of AMD will clarify whether kallistatin could be used as a biomarker of AMD. Recently, aqueous Wnt signaling modulators, Wnt inhibitory factor 1 (WIF-1) and Dickkopf 3 (DKK-3) are reported significantly higher in neovascular AMD patients, the high WIF-1 levels are also associated with disrupted length of photoreceptor inner and outer segments [56]. This finding supports the implication of Wnt signaling in photoreceptor integrity and AMD.

In summary, the balance of Wnt signaling activation may disturb homeostasis and allostasis in the normal aging retina, which could be linked to AMD pathogenesis due to allostatic overload. The activation of canonical Wnt signaling might contribute to the focal retinal degeneration of mouse models with *Ccl2* and *Cx3cr1* deficiency, and intravitreal anti-LRP6 antibody therapy might be beneficial via deactivation of the canonical Wnt pathway.

## Conclusion

This study reported strong evidence of Wnt signaling involvement in AMD. A beneficial effect was found on retina lesions by intravitreal administration of anti-LRP6 antibody to deactivate the canonical Wnt pathway in two related mouse models with focal retinal degeneration mimicking certain features of human AMD.

## Additional files

10.1186/s12967-015-0683-x RPE lesion in *CCL2* ^*-/-*^ *CX3CR1* ^*gfp/gfp*^ mice. Three months old *CCL2* ^*-/-*^ *CX3CR1* ^*gfp/gfp*^ mice were exposed to blue light (460 nm, 8 h/day) for 6 months. RPE/choroid flatmounts were stained with rhodamine-phalloidin (Red, for F-actin) and observed by confocal microscopy. The image shows disorganised F-actin distribution (sign of RPE damage) and infiltration of GFP+ macrophages / microglial cells at the lesion site. Scale bar = 50 mm. **Figure S2.** Western blot analysis of LRP6 expression. The anti-LRP6 antibody used in immunohistochemistry was tested in cell lysate from human retinal pigment epithelia cell line ARPE19. Proteins were separated by 8 % SDS-PAGE gel. **Figure S3.** Correlation analysis between the age and plasma kallistatin level in AMD and control subjects. There is no correlation between the age and plasma kallistatin level in either AMD or control subjects. **Figure S4.** **A**–**C**: Microarray of 92 Wnt genes in 3 pairs of eyes (3 mice). The differentiated mRNA expression shows no differences in the retina between *Ccl2* ^*-/-*^ */Cx3cr1* ^*-/-*^ */rd8* and B6N mice. **Figure S5.** ERG of *Ccl2* ^*-/-*^ */Cx3cr1* ^*-/-*^ */rd8* and B6N. The b wave amplitudes are significantly lower in B6N retina compared to *Ccl2* ^*-/-*^ */Cx3cr1* ^*-/-*^ */rd8* retina under light adapted, UV or green light, indicating damages in bipolar, S cone function.
10.1186/s12967-015-0683-x Gene list of Wnt mRNA Microarray. **Table S2**. The cluster of AMD cases with high kallistatin level.

## Abbreviations

Wnt: wingless-type MMTV integration site
LRP6: lipoprotein receptor-related protein
A2E: ([2,6-dimethyl-8-(2,6,6-trimethyl-1-cyclohexen-1-yl)-1E,3E,5E,7E-octatetra-enyl]-1-(2-hydroxyethyl)-4-[4-methyl-6(2,6,6-trimethyl-1-cyclohexen-1-yl) 1E,3E,5E,7E-hexatrienyl]-pyridinium)
