# Supplementary figures and images for: Wnt signaling in age-related macular degeneration: human macular tissue and mouse model
Source: J Transl Med. 2015 Oct 17;13:330. doi: 10.1186/s12967-015-0683-x (PMC4609061; doi:10.1186/s12967-015-0683-x)

Figure S1

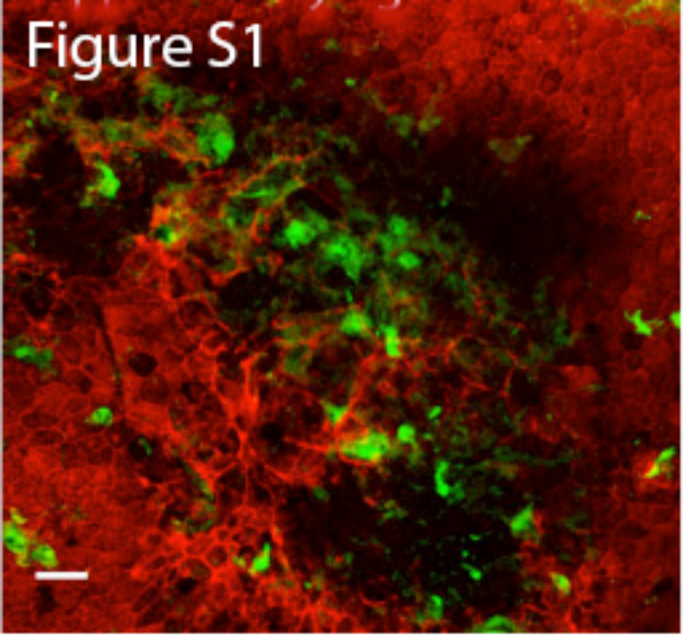

Figure S2

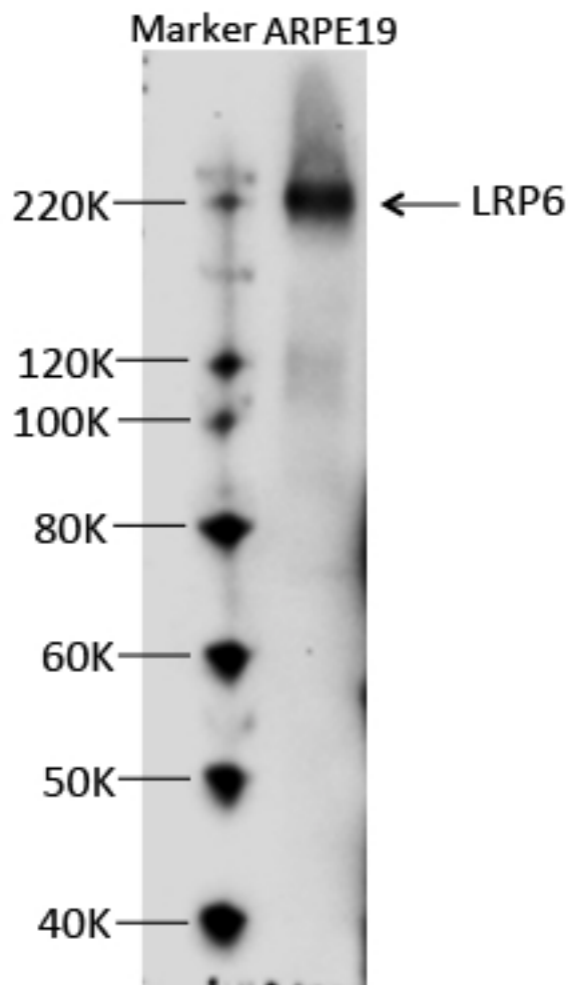

Figure S3

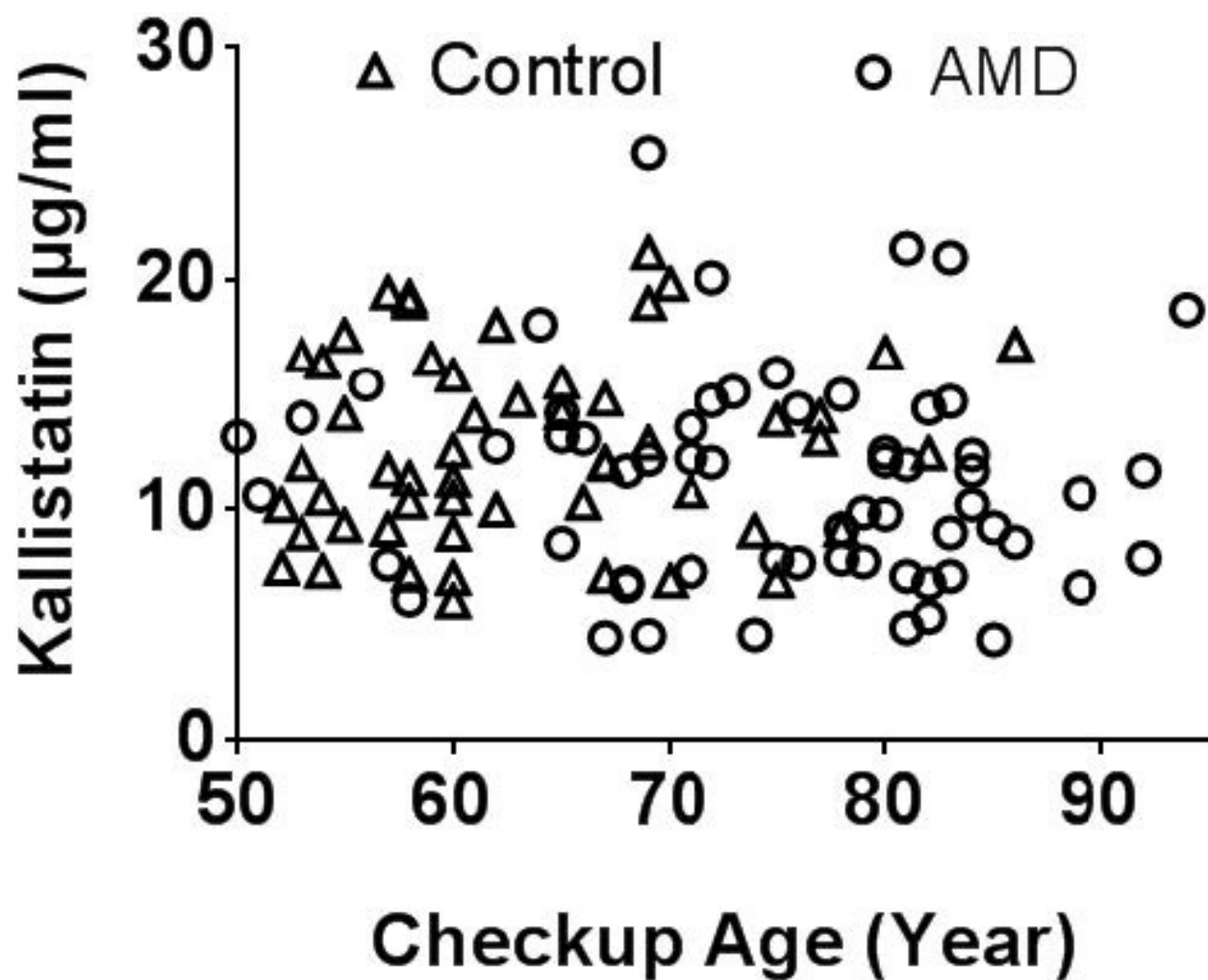

Figure S4A

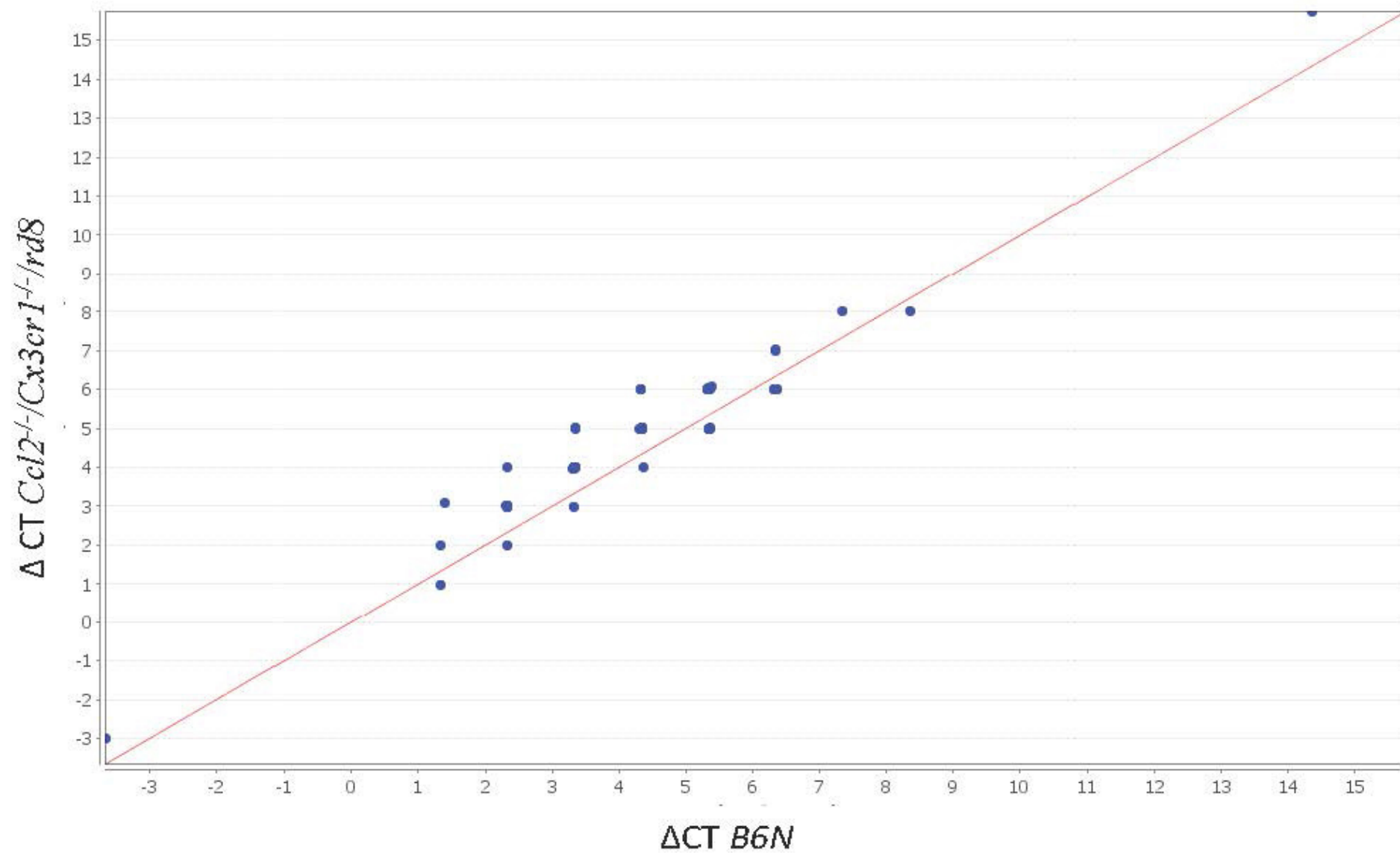

Figure S4B

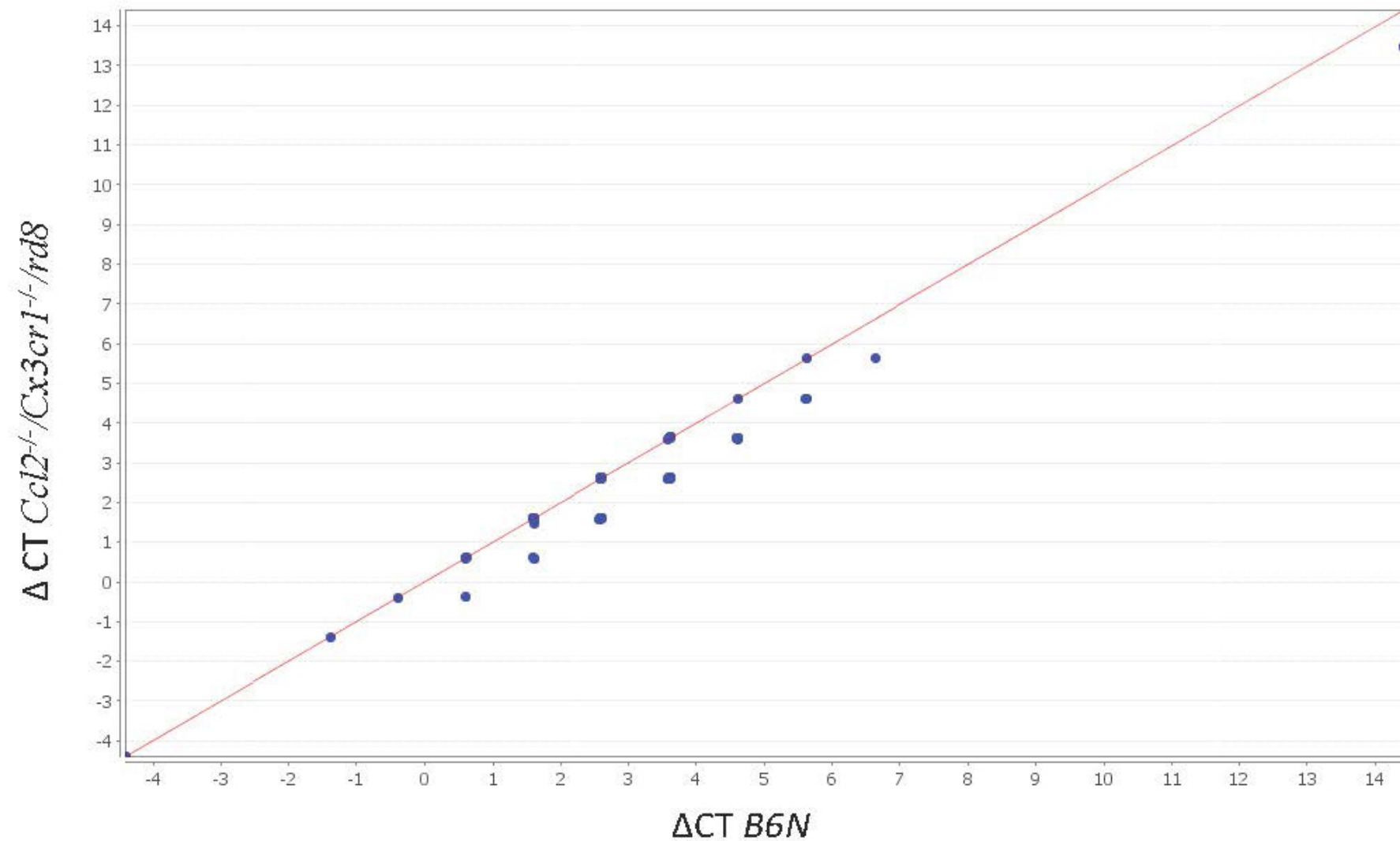

Figure S4C

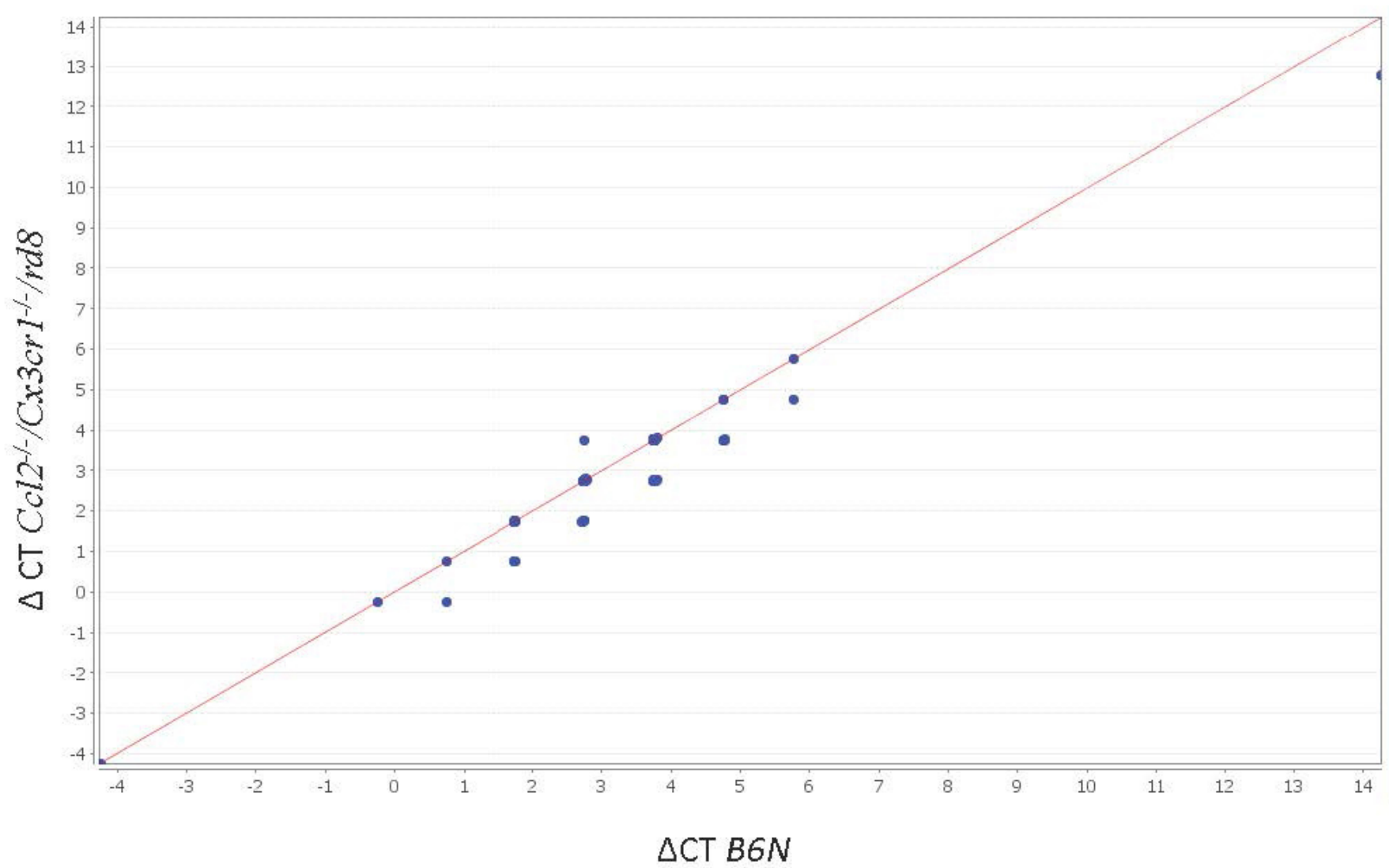

Figure S5

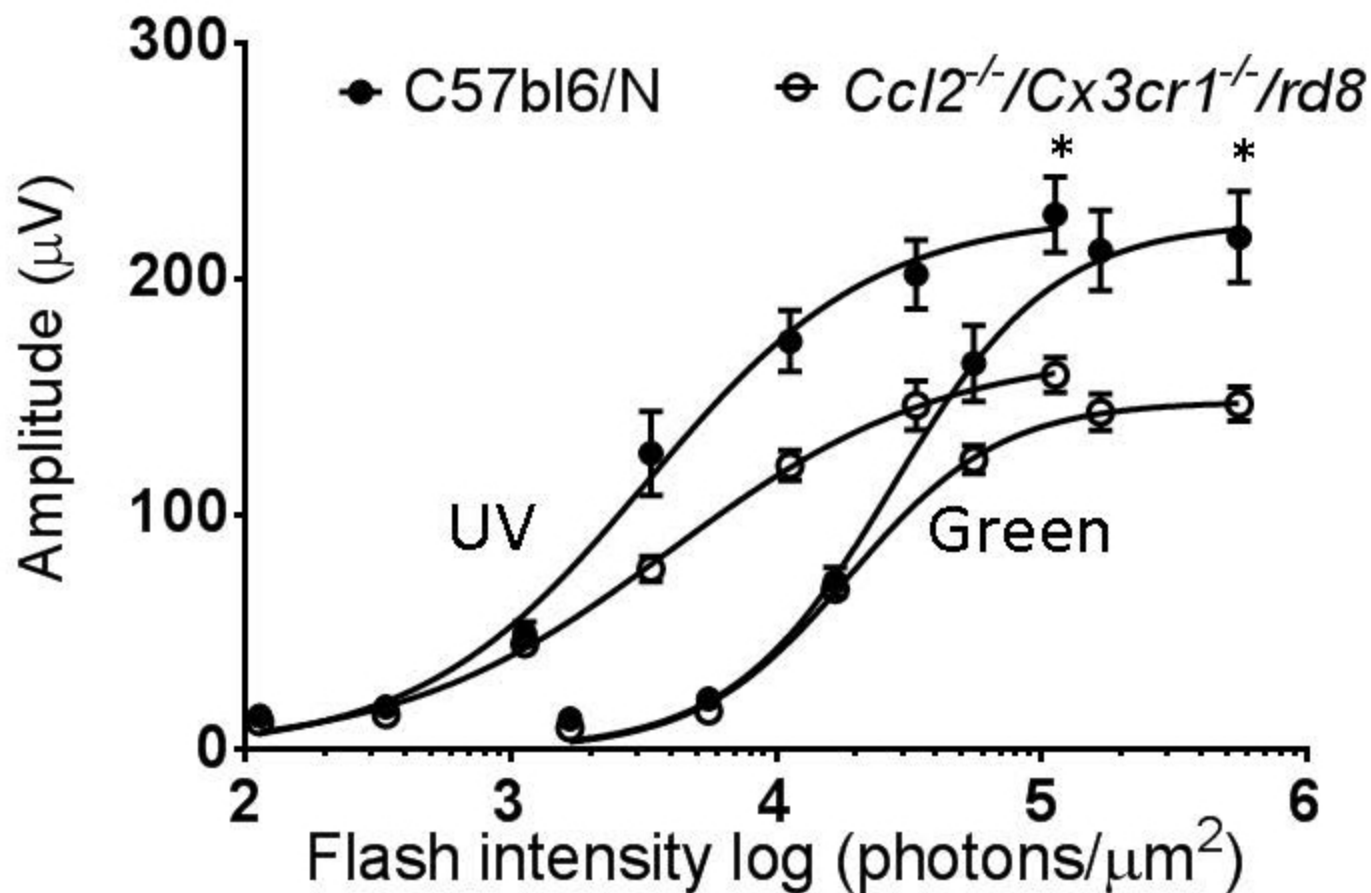

Supplement: Supplementary file 1 — 10.1186/s12967-015-0683-x RPE lesion in CCL2 -/- CX3CR1 gfp/gfp mice. Three months old CCL2 -/- CX3CR1 gfp/gfp mice were exposed to blue light (460 nm, 8 h/day) for 6 months. RPE/choroid flatmounts were stained with rhodamine-phalloidin (Red, for F-actin) and observed by confocal microscopy. The image shows disorganised F-actin distribution (sign of RPE damage) and infiltration of GFP+ macrophages / microglial cells at the lesion site. Scale bar = 50 mm. Figure S2. Western blot analysis of LRP6 expression. The anti-LRP6 antibody used in immunohistochemistry was tested in cell lysate from human retinal pigment epithelia cell line ARPE19. Proteins were separated by 8 % SDS-PAGE gel. Figure S3. Correlation analysis between the age and plasma kallistatin level in AMD and control subjects. There is no correlation between the age and plasma kallistatin level in either AMD or control subjects. Figure S4. A–C: Microarray of 92 Wnt genes in 3 pairs of eyes (3 mice). The differentiated mRNA expression shows no differences in the retina between Ccl2 -/- /Cx3cr1 -/- /rd8 and B6N mice. Figure S5. ERG of Ccl2 -/- /Cx3cr1 -/- /rd8 and B6N. The b wave amplitudes are significantly lower in B6N retina compared to Ccl2 -/- /Cx3cr1 -/- /rd8 retina under light adapted, UV or green light, indicating damages in bipolar, S cone function. [file 12967_2015_683_MOESM1_ESM.pdf]
