# Supplementary material for: Wnt signaling in age-related macular degeneration: human macular tissue and mouse model
Source: J Transl Med. 2015 Oct 17;13:330. doi: 10.1186/s12967-015-0683-x (PMC4609061; doi:10.1186/s12967-015-0683-x)
Supplement: Supplementary file 2 — 10.1186/s12967-015-0683-x Gene list of Wnt mRNA Microarray. Table S2. The cluster of AMD cases with high kallistatin level. [file 12967_2015_683_MOESM2_ESM.docx]

| Aes | Apc | Axin1 | Bcl9 | Btrc | Ctnnbip1 | Ccnd1 | Ccnd2 | Ccnd3 | Csnk1a1 | Csnk1d | Csnk2a1 |
| --- | --- | --- | --- | --- | --- | --- | --- | --- | --- | --- | --- |
| Ctbp1 | Ctbp2 | Ctnnb1 | Daam1 | Dixdc1 | Dkk1 | Dvl1 | Dvl2 | Ep300 | Fbxw11 | Fbxw2 | Fbxw4 |
| Fgf4 | Fosl1 | Foxn1 | Frat1 | Frzb | Fshb | Fzd1 | Fzd2 | Fzd3 | Fzd4 | Fzd5 | Fzd6 |
| Fzd7 | Fzd8 | Gsk3b | Jun | Kremen1 | Lef1 | Lrp5 | Lrp6 | Myc | Nkd1 | Nlk | Pitx2 |
| Porcn | Ppp2ca | Ppp2r1a | Ppp2r5d | Pygo1 | Rhou | Senp2 | Sfrp1 | Sfrp2 | Sfrp4 | Slc9a3r1 | Sox17 |
| T | Tcf7l1 | Tcf7 | Tle1 | Tle2 | Wif1 | Wisp1 | Wnt1 | Wnt10a | Wnt11 | Wnt16 | Wnt2 |
| Wnt2b | Wnt3 | Wnt3a | Wnt4 | Wnt5a | Wnt5b | Wnt6 | Wnt7a | Wnt7b | Wnt8a | Wnt8b | Wnt9a |
| Gusb | Hprt | Hsp90ab1 | Gapdh | Actb | MGDC | RTC | RTC | RTC | PPC | PPC | PPC |

Table S1: Gene list of Wnt mRNA Microarray

Table S2: The cluster of AMD cases with high kallistatin level

| Sample | Date of collection | Checkup Age | Gender | AMD Grade | Wet/Dry | Smoker | Cataract | Kallistatin (μg/ml) |
| --- | --- | --- | --- | --- | --- | --- | --- | --- |
| 1 | 09/27/10 | 64 | M | 3 | W | 1 | NA | 18.04 |
| 2 | 04/30/12 | 94 | F | 3 | W | 1 | NA | 18.70 |
| 3 | 06/21/10 | 72 | M | 3 | W | 1 | No | 20.08 |
| 4 | 11/13/09 | 83 | F | 3 | W | 0 | No | 20.95 |
| 5 | 08/28/09 | 81 | F | 3 | D | 0 | yes | 21.36 |
| 6 | 07/14/10 | 69 | M | 3 | W | 1 | yes | 25.48 |
